# *In vivo* assessment of pravastatin efficacy in an L-NAME rat model of preeclampsia: uncoupling of functional and structural recovery via combined photoacoustic and ultrasound imaging

**DOI:** 10.3389/fphar.2026.1782936

**Published:** 2026-05-19

**Authors:** Bo Zhang, Houqing Pang, Haibo Yang, Hong Luo

**Affiliations:** 1 Department of Ultrasound, West China Second Hospital, Sichuan University, Chengdu, China; 2 Key Laboratory of Birth Defects and Related Diseases of Women and Children (Sichuan University), Ministry of Education, Chengdu, China

**Keywords:** contrast-enhanced ultrasound, drug screening, pharmacodynamics, photoacoustic imaging, pravastatin, preeclampsia

## Abstract

**Background:**

Preeclampsia (PE) drug development is often hindered by the lack of sensitive, non-invasive tools for monitoring longitudinal placental responses *in vivo*. Traditional metrics, such as uterine artery resistance index (UtA-RI), primarily reflect downstream vascular resistance and may overlook subtle improvements in microvascular perfusion and oxygenation. In this study, using pravastatin as a model therapeutic agent, we aimed to evaluate the utility of a longitudinal multimodal imaging platform—integrating pulse-wave Doppler, microvascular flow imaging (MV-flow), contrast-enhanced ultrasound (CEUS), and photoacoustic imaging (PAI)—to capture the temporal dynamics of placental recovery in an Nω-nitro-L-arginine methyl ester (L-NAME)-induced PE rat model. By adhering to the 3Rs principles, we sought to assess these imaging biomarkers as potential indicators of therapeutic efficacy.

**Methods:**

PE was induced in Sprague–Dawley rats via L-NAME (GD7–18), with pravastatin or saline administered from GD10–18. Longitudinal imaging was performed on GD14, 16, and 18 to assess oxygenation (sO_2_), microvascular blood volume (WiAUC), microvascular density (VI), and macroscopic arterial resistance (UtA-RI). Exploratory metrics, including longitudinal recovery (β) and functional recovery index (FRI), were calculated to characterize the therapeutic trajectory and extent.

**Results:**

Pravastatin attenuated maternal hypertension and proteinuria. Crucially, imaging revealed a distinct temporal mismatch: functional markers (sO_2_ and WiAUC) improved significantly as early as GD14 and GD16, respectively. Conversely, structural and hemodynamic indices (VI and UtA-RI) exhibited a therapeutic lag, improving only at GD18. Consequently, the FRI for functional markers (sO_2_: 92.7%; WiAUC: 88.1%) significantly exceeded that of macroscopic arterial resistance (UtA-RI: 43.9%). Histology (HIF-1α/CD31) corroborated these phenotypes.

**Discussion:**

Functional biomarkers (sO_2_, WiAUC) demonstrate potential for earlier detection of efficacy compared to structural indices. This observed temporal dissociation is consistent with the hypothesis that pravastatin may mobilize a “functional reserve” prior to architectural remodeling. Although reflecting the specific vascular tone restoration of the L-NAME model, this study demonstrates the feasibility of this multimodal framework for characterizing the spatiotemporal pharmacodynamics of novel placental therapeutics.

## 1 Introduction

Preeclampsia (PE) remains a significant cause of maternal and perinatal morbidity, characterized by placental ischemia and widespread maternal endothelial dysfunction (Chaiworapongsa et al., 2014; Rana et al., 2019). While the etiology is multifactorial, the failure of physiological spiral artery remodeling leads to reduced uteroplacental perfusion, triggering a cascade of oxidative stress and angiogenic imbalance (Phipps et al., 2019). Currently, delivery remains the only definitive cure. Consequently, drug repurposing strategies, such as the use of pravastatin, are being investigated as potential interventions to restore endothelial function and prolong gestation (Costantine et al., 2013; Ahmed and Ramma, 2015).

However, the precise spatiotemporal pharmacodynamics of such interventions remain poorly characterized. In clinical obstetrics, placental monitoring relies predominantly on the uterine artery resistance index (UtA-RI) (O’Gorman et al., 2017). Because UtA-RI is primarily dictated by the anatomical extent of spiral artery remodeling, it serves as a valuable indicator of macrovascular structure but inherently lacks sensitivity to tissue-level microcirculatory physiology (Prefumo et al., 2004). This structural nature underpins a recognized clinical disconnect: antihypertensive therapies can successfully normalize systemic function (e.g., maternal blood pressure) yet frequently fail to reverse an established high-resistance UtA-RI (Khalil et al., 2010). Consequently, an exclusive reliance on these refractory structural indices may obscure early therapeutic responses. Building upon this disconnect, we hypothesize a ‘therapeutic lag’ during pharmacological intervention, wherein the restoration of local microcirculatory function (oxygenation and perfusion) temporally precedes the normalization of macroscopic vascular architecture.

To address this challenge, it is necessary to explore multimodal imaging strategies that characterize the oxygenation, perfusion, and microvascular density dynamics of the placenta beyond macroscopic arterial resistance. In this study, we integrated three complementary modalities: (1) Photoacoustic imaging (PAI), which distinguishes oxy- and deoxy-hemoglobin to specifically quantify hemoglobin oxygen saturation (sO_2_), offering a proxy for tissue oxygenation status (Arthuis et al., 2017; Yamaleyeva et al., 2017); (2) Contrast-enhanced ultrasound (CEUS), which utilizes microbubbles to visualize capillary perfusion independent of flow velocity, reflecting functional perfusion (Roberts et al., 2016; Jain et al., 2025); and (3) Microvascular flow imaging (MV-flow), an advanced Doppler technique that suppresses tissue motion artifacts to depict microvascular density (Aziz et al., 2022).

To investigate these spatiotemporal kinetics, we utilized the Nω-nitro-L-arginine methyl ester (L-NAME)-induced rat model of PE. By systemically inhibiting nitric oxide synthase (NOS), this model recapitulates key features of endothelial dysfunction, systemic vasoconstriction, and placental hypoperfusion characteristic of the human preeclamptic phenotype, providing a clinically relevant substrate to evaluate endothelial-protective therapeutics like pravastatin (Bakrania et al., 2022). To quantitatively capture the dynamics of the therapeutic response, we introduced two exploratory metrics: the Longitudinal Recovery Slope to measure the rate of recovery, and the Functional Recovery Index (FRI) to quantify the degree of normalization. By integrating this preclinical model with longitudinal multimodal imaging, this study aims to: (1) elucidate the spatiotemporal dissociation between microcirculatory functional restoration and macrovascular hemodynamic recovery; (2) validate these *in vivo* imaging findings with *ex vivo* histological markers; and (3) explore the feasibility of the Slope and FRI metrics for assessing therapeutic efficacy. Ultimately, we hope this combined approach offers a non-invasive, translational perspective for evaluating the spatiotemporal pharmacodynamics of novel placental therapeutics.

## 2 Methods

### 2.1 Animal model establishment and therapeutic regimen

Ethical approval was obtained from the Biomedical Ethics Committee of West China Second Hospital, Sichuan University (No. 2023121). The experimental protocol was conducted in accordance with the ARRIVE guidelines and the 3R principles to prioritize animal welfare.

Female Sprague–Dawley rats (aged 14–16 weeks, weighing 230–250 g) were purchased from Huafukang Biotechnology Co., Ltd. (Beijing, China) and housed under standard specific-pathogen-free (SPF) conditions (12-h light/dark cycle, 22 °C ± 2 °C) with *ad libitum* access to food and water. Following overnight cohabitation, the presence of a vaginal plug was designated as gestational day (GD) 0. Consistent with the principle of Reduction, pregnancy was confirmed on GD7 via high-frequency ultrasound screening (Supplementary Figure S1). This early screening allowed for the exclusion of non-pregnant rats (*n =*24) prior to group allocation, thereby optimizing the sample size. Dams with confirmed viable gestational sacs were randomly allocated into three groups (*n* = 8 per group): control group, PE group, and PE + pravastatin (pravastatin treatment group) (Supplementary Figure S2).

**FIGURE 1:**
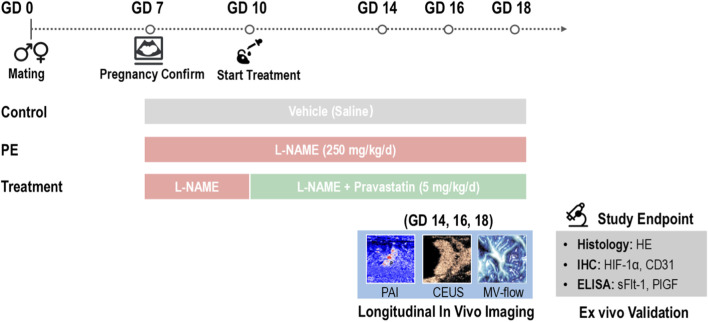
Schematic representation of the experimental protocol and longitudinal multimodal imaging design. The study timeline illustrates the induction of the preeclampsia-like phenotype using L-NAME (GD7–GD18), the therapeutic window of pravastatin intervention (GD10–GD18), and the time points for longitudinal imaging (GD14, 16, and 18).

To induce the PE-like phenotype, L-NAME (Sigma-Aldrich, St. Louis, MO, USA; 250 mg/kg/day) was administered via intraperitoneal (i.p.) injection from GD7 through GD18 (Soobryan et al., 2017; Yang et al., 2020). To evaluate the efficacy of intervention following the initiation of vascular insult, the Treatment group received pravastatin (Sigma-Aldrich, St. Louis, MO, USA; 5 mg/kg/day) intraperitoneally starting from GD10 (representing a mid-gestation therapeutic window) and continued daily until GD18. The control group received equivolume saline vehicle injections daily throughout the study period (GD7–GD18). The detailed experimental protocol is summarized in Figure 1.

To validate the model and monitor therapeutic efficacy, systolic blood pressure (SBP) was measured longitudinally at key time points (GD7, 10, 13, and 18) using a non-invasive tail-cuff system (Medlab; Nanjing Calvin Biotechnology Co., Ltd., Nanjing, China). Rats were warmed to 37 °C for 10 min and acclimatized to the restraint device prior to measurements to minimize stress-induced variability. Additionally, rats were placed in metabolic cages to collect 24-h urine samples for proteinuria assessment at the same time points. Maternal body weight was recorded daily. Fetal viability and biometric growth were longitudinally monitored via HFUS throughout the experimental period (Supplementary Figure S3).

### 2.2 Longitudinal multimodal imaging platform

A non-invasive longitudinal imaging strategy was employed to monitor placental morphology and function within the same subject on GD14, 16, and 18. This approach minimized inter-individual variability and reduced animal usage, aligning with the 3R principles mentioned above. Prior to each session, anesthesia was induced with 3% isoflurane and maintained at 1.5%–2% in 100% oxygen (flow rate: 1.0 L/min) via a nose cone following abdominal depilation. The rats were placed on a heated stage, and the total anesthesia duration was strictly limited to approximately 30 min to maintain hemodynamic stability.

A sequential imaging protocol was performed under continuous anesthesia. First, oxygenation data were captured using a Vevo LAZR photoacoustic system (Fujifilm VisualSonics, Toronto, Canada) utilizing a hybrid linear transducer (LZ-250). Subsequently, the animal was transferred to the Samsung ultrasound system (Samsung Medison, Seoul, Korea; Probe: LA2-14A) for morphological, hemodynamic, and perfusion assessments. This sequence (PAI prior to CEUS) was chosen to prevent highly scattering exogenous microbubbles from interfering with the photoacoustic assessment of endogenous oxygenation. To minimize time-dependent physiological drift (e.g., isoflurane-induced vasodilation) during imaging, the temporal workflow was standardized across all animals, and baseline physiological stability was maintained using a 37 °C heated platform with continuous heart rate monitoring.

At the initial GD14 session, a single representative placenta was selected per dam for longitudinal monitoring. Selection was restricted to placentas with live fetuses located near the midline to ensure consistent acoustic and optical access across all sessions. The umbilical cord insertion served as a fixed landmark for cross-platform co-registration. Both probes were manipulated to visualize anatomically consistent maximal sagittal planes, defined by the central canal and maximal placental width, ensuring that metrics were derived from comparable regions.

### 2.3 Placenta morphological and hemodynamic assessment

#### 2.3.1 Morphological and macro-vascular assessment

**FIGURE 2:**
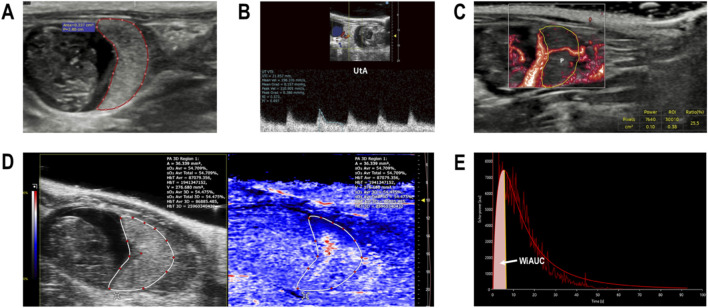
Representative images of longitudinal multimodal placental assessment. **(A)** B-mode ultrasound for the morphological assessment of maximal placental cross-sectional area (red outline). **(B)** Uterine artery (UtA) pulse-wave Doppler waveforms used for resistance index (RI) calculation. **(C)** MV-flow imaging visualizing microvascular flow signals (vascular index, VI). **(D)** Photoacoustic imaging (PAI) map showing the distribution of hemoglobin oxygen saturation (sO_2_). **(E)** Representative contrast-enhanced ultrasound (CEUS) time-intensity curve (TIC). Red line: fitted model; shaded area: wash-in area under the curve (WiAUC), serving as a surrogate for relative microvascular blood volume.

Analysis was conducted by a blinded sonographer. Placental maximal cross-sectional area was measured using B-mode ultrasound (Figure 2A). For hemodynamic assessment, the Uterine Artery (UtA) was interrogated using Pulse-Wave Doppler with a 1.0 mm sample volume and angle correction (<60°). The resistance index (UtA-RI) was calculated using the standard formula: RI = (PSV − EDV)/PSV, where PSV is the peak systolic velocity and EDV is the end-diastolic velocity. A decrease in UtA-RI indicates reduced downstream vascular resistance. Measurements were derived from the average of 3–5 consecutive cardiac cycles (Figure 2B).

#### 2.3.2 Microvascular density imaging (MV-flow)

To visualize slow-velocity microvascular signals, MV-flow mode was applied with high-sensitivity settings (PRF: 0.13 kHz; Wall Filter: Low). The Vascular Index (VI) was quantified within a region of interest (ROI) delimiting the entire placental parenchyma (Figure 2C). VI was defined as the percentage of color flow signals relative to the total ROI area, serving as a surrogate marker for placental microvascular density. A constant color threshold was utilized across all subjects to ensure consistent quantification.

### 2.4 Placenta functional oxygenation and perfusion assessment

#### 2.4.1 Oxygenation imaging (PAI)

Images were acquired in Oxyhemo mode using a tunable Nd:YAG-pumped OPO laser with dual-wavelength excitation (750/850 nm). Optical fluence was maintained <20 mJ/cm^2^ (within ANSI safety limits), and degassed coupling gel was utilized to minimize acoustic artifacts. To minimize optical attenuation variations, the PAI transducer depth was fixed at approximately 9 mm from the maternal skin surface across all animals and longitudinal sessions. Pixel-wise oxygen saturation (sO_2_) maps were derived via linear spectral unmixing based on the differential optical absorption of oxy-hemoglobin (HbO_2_) and deoxy-hemoglobin (Hb) (Figure 2D). To mitigate potential depth-dependent attenuation, quantitative analysis focused on relative changes within the placental parenchyma, excluding large vessels. Mean sO_2_ values were extracted from ROIs defined on co-registered anatomical planes.

#### 2.4.2 Perfusion imaging (CEUS)

Performed on the Samsung ultrasound platform post-PAI. A standardized bolus of SonoVue (1.0 mL/kg, i.v.) was injected via the tail vein, immediately followed by a saline flush to ensure consistent contrast delivery. Real-time acquisition was maintained at a low mechanical index (MI = 0.07) to prevent microbubble destruction. Quantitative analysis was performed using VueBox software with motion correction. Specifically, the Wash-in Area Under the Curve (WiAUC) was calculated from Time-Intensity Curves (TICs) (Figure 2E). Correlating theoretically with the perfused capillary cross-sectional area, WiAUC serves as a velocity-independent index of relative microvascular blood volume available for maternal-fetal exchange.

### 2.5 Exploratory quantitative pharmacodynamic metrics

To complement static endpoint comparisons and quantitatively characterize the temporal kinetics of the therapeutic response to pravastatin, two exploratory metrics were derived.

#### 2.5.1 Longitudinal recovery slope (β)

To characterize the general trajectory of the therapeutic response, a trend analysis was performed on the time-series data (GD14, 16, and 18) for each subject. Given the limited number of time points, a linear fit was applied as a simplified exploratory model to capture the direction and magnitude of recovery. The slope coefficient (β) was directionally adjusted to ensure consistent interpretability across physiologically distinct parameters. Specifically, the raw slope was utilized for indices where an increase represents recovery (e.g., sO_2_, WiAUC, and VI), whereas the slope was inverted (-β) for indices where a decrease indicates improvement (e.g., UtA-RI). Consequently, a positive adjusted β consistently indicates the velocity of restoration toward the healthy phenotype, with the magnitude representing the overall rate of improvement.

#### 2.5.2 Functional recovery index (FRI)

To facilitate the comparison of heterogeneous modalities with different physical units, a normalized recovery index was calculated at the study endpoint (GD18). The FRI quantifies the extent to which the treatment restored a specific parameter relative to the disease baseline, defined as:

$$FRI=\frac{X_{\dot{i}}-{\bar{X}}_{PE}}{{\bar{X}}_{Control}-{\bar{X}}_{PE}}\times 100\%$$

where ${\;X}_{\dot{i}}$ represents the individual value of a treated animal, and ${\bar{X}}_{Control}$ and ${\bar{X}}_{PE}$ represent the group mean values of the control and PE groups, respectively. On this normalization scale, 100% indicates complete normalization to the healthy control level, 0% indicates no improvement over the untreated PE phenotype, and negative values suggest further deterioration relative to the disease model.

### 2.6 Histopathological and biochemical corroboration

At the study endpoint (GD18), rats were deeply anesthetized with 5% isoflurane. Blood was collected via cardiac puncture for serum analysis, followed by vital organ excision to ensure euthanasia. Fetal and placental wet weights were recorded. Serum sFlt-1 and PlGF concentrations were quantified to assess systemic angiogenic status using commercial enzyme-linked immunosorbent assay (ELISA) kits (Rat sFlt-1 R1 ELISA Kit, Cat. No. ZC-55018; and Rat PLGF ELISA Kit, Cat. No. ZC-37237; both from Shanghai Zhuocai Biotechnology Co., Ltd., Shanghai, China). All assays were performed according to the manufacturer’s protocols, and the absorbance was measured at 450 nm using a microplate reader.

Placentas were harvested, paraffin-embedded, and sectioned (4-μm) along the midline sagittal plane to approximate the anatomical region assessed during *in vivo* imaging. Immunohistochemistry (IHC) was performed to evaluate tissue hypoxia and microvascular density. The primary antibodies utilized were: rabbit polyclonal anti-HIF-1α (1:50, Cat. No. BS-0737R, Bioss, Beijing, China) and rabbit monoclonal anti-CD31 (1:1,000, clone EPR17259, Cat. No. ab182981, Abcam, Cambridge, MA, USA). To assess spiral artery remodeling, adjacent supplemental sections were stained for Cytokeratin 7 (CK7, marking trophoblasts; rabbit polyclonal, 1:500, Cat. No. 15539-1-AP, Proteintech, Wuhan, China) and α-smooth muscle actin (α-SMA, marking vascular smooth muscle; rabbit polyclonal, 1:200, Cat. No. ab5694, Abcam, Cambridge, MA, USA).

All slides were digitized and quantified using the HALO platform via a blinded, automated Whole Slide Imaging (WSI) workflow. Specific Regions of Interest (ROIs) were defined within the placental labyrinth zone for HIF-1α/CD31 and the decidual/junctional zone for remodeling markers. The Positivity Area Percentage was utilized to provide *ex vivo* biological corroboration for the functional and structural alterations observed *in vivo*.

### 2.7 Statistical analysis

Statistical analyses were performed using GraphPad Prism 9.0. Data normality and homoscedasticity were verified using the Shapiro-Wilk and Brown-Forsythe tests, respectively. Continuous variables are expressed as mean ± SEM. Longitudinal data (GD14–18) were analyzed via Two-way Repeated Measures ANOVA (Treatment × Time) followed by Bonferroni *post hoc* tests to adjust for multiple comparisons. For the exploratory pharmacodynamic metrics, the Longitudinal Recovery Slopes (β) were compared using Analysis of Covariance (ANCOVA) to test for differences in regression coefficients between groups. Differences in FRI values across imaging modalities were assessed via One-way ANOVA with Tukey’s *post hoc* test. To explore potential associations between imaging metrics and physiological outcomes (fetal weight), simple linear regression analysis was performed. To evaluate the correlation between *in vivo* and *ex vivo* measurements, Pearson’s correlation coefficient (r) was calculated across all experimental subjects. No outliers were excluded from the analysis. A two-tailed *P <* 0.05 was considered statistically significant.

## 3 Results

### 3.1 Verification of model establishment and systemic therapeutic efficacy

**FIGURE 3:**
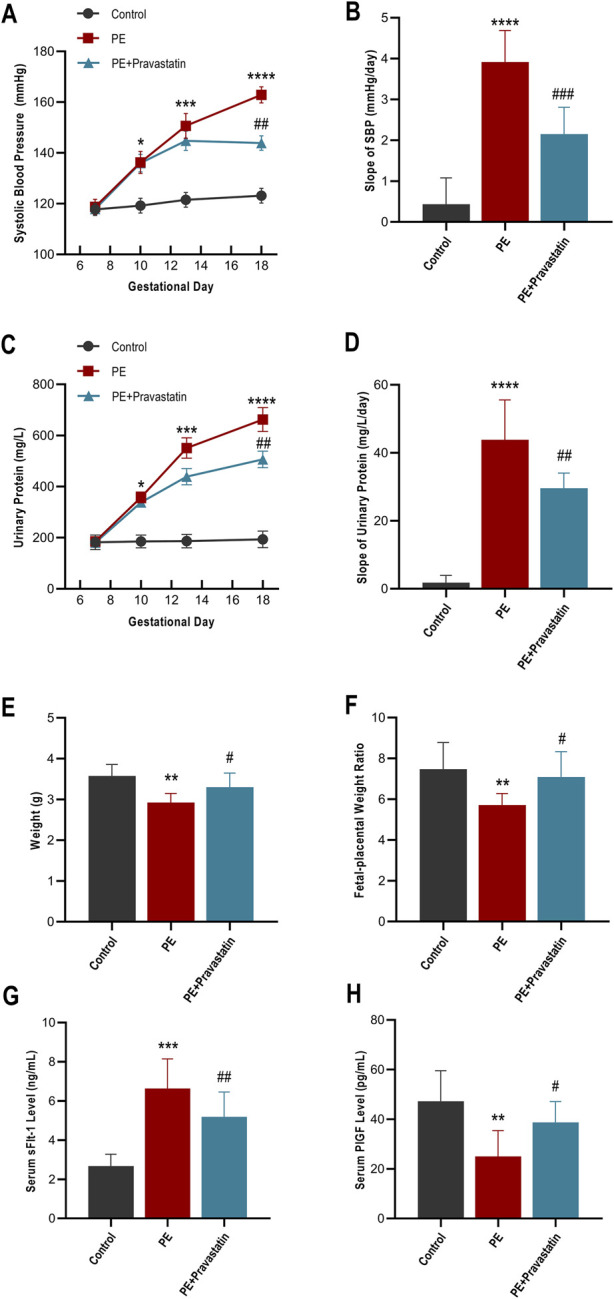
Evaluation of systemic therapeutic response and angiogenic profile. **(A–D)** Longitudinal trajectories and slope analysis of systolic blood pressure (SBP) and 24-h urinary protein levels. **(E,F)** Fetal weight and fetal-placental weight ratio recorded at GD18. **(G,H)** Maternal serum concentrations of sFlt-1 and PlGF (ELISA) at GD18. Data are presented as mean ± SEM. *P < 0.05, **P < 0.01; ***P < 0.001, ****P < 0.0001 vs. control; #*P <* 0.05, ##*P* < 0.01, ###*P <* 0.001 vs. PE.

Longitudinal monitoring confirmed the expected phenotypic alterations following L-NAME administration, verifying the establishment of the PE model. The PE group exhibited a progressive and significant rise in SBP and proteinuria compared to controls. Pravastatin intervention, initiated from GD10, effectively attenuated this progression. By the study endpoint (GD18), SBP in the Treatment group was significantly reduced compared to the PE group (143.9 ± 8.1 vs. 162.9 ± 9.0 mmHg, *P <* 0.01). Similarly, 24-h urinary protein levels were markedly lower in the Treatment group (506.3 ± 92.8 vs. 662.6 ± 131.3 mg/L, *P <* 0.01). Exploratory slope analysis further illustrated the attenuation of disease progression trends: the Treatment group displayed significantly flatter trajectories for both SBP (Slope: 2.15 ± 0.23 vs. 3.91 ± 0.27 mmHg/day, *P <* 0.001) and proteinuria (Slope: 29.55 ± 1.59 vs. 43.84 ± 4.15 mg/L/day, *P <* 0.01) (Figures 3A–D).

Correspondingly, endpoint assessments indicated that the pathological impact on fetal development was significantly mitigated. While the PE group exhibited significant fetal growth restriction (FGR) and increased resorption, fetal weight at GD18 in the pravastatin treatment group was significantly increased compared to the PE group (3.30 ± 0.35 g vs. 2.93 ± 0.23 g, *P <* 0.05). Notably, this fetal weight improvement was associated with the preservation of placental efficiency, as evidenced by the significantly improved fetal-placental weight ratio in the Treatment group (7.1 ± 1.2 vs. 5.7 ± 0.6, *P <* 0.05) (Figures 3E,F).

Regarding systemic angiogenic markers, this phenotypic improvement coincided with a shift in angiogenic balance. ELISA analysis of maternal serum at GD18 showed that pravastatin significantly attenuated the L-NAME-induced imbalance, downregulating anti-angiogenic sFlt-1 and upregulating pro-angiogenic PlGF relative to the untreated PE group (Figures 3G,H).

### 3.2 Delayed response in placental morphology and hemodynamics

#### 3.2.1 Placental morphology

B-mode ultrasound monitoring showed no significant therapeutic impact on placental dimensions. The placental maximal cross-sectional area in the Treatment group did not differ significantly from the PE group throughout the study period (*P > 0.05*).

#### 3.2.2 Macroscopic arterial resistance (UtA-RI)

**FIGURE 4:**
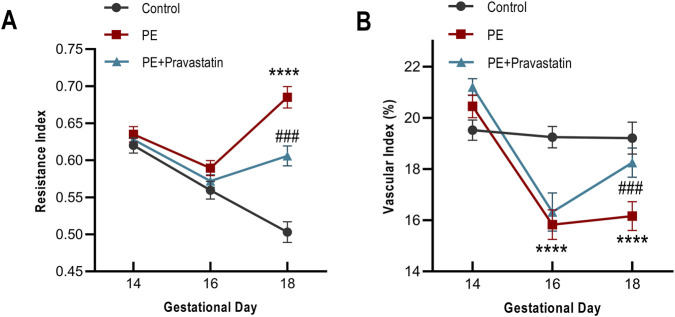
Longitudinal assessment of placental vascular resistance and microvascular density. **(A)** Temporal profiles of the uterine artery resistance index (UtA-RI) from GD14 to GD18. **(B)** Quantification of microvascular density (VI) using MV-flow imaging. Data are presented as mean ± SEM. ****P < 0.0001 vs. control; ###*P <* 0.001 vs. PE.

Significant alterations in uterine artery hemodynamics were not observed in the early phase. At GD14 and GD16, the UtA-RI in the Treatment group was comparable to that of the PE group. A significant reduction was only detected at the study endpoint (GD18), where UtA-RI values in the Treatment group were significantly lower than those in the untreated PE group (*P < 0.001*). Despite this late-stage improvement, the values did not fully return to control levels, resulting in an FRI of 43.9% ± 4.4% (Figure 4A).

#### 3.2.3 Microvascular density (MV-flow VI)

The microvascular density (VI) showed a similar trend of delayed recovery. From GD14 to GD16, the VI in the Treatment group decreased alongside the PE group (*P > 0.05*). A statistical difference emerged only at GD18 (*P < 0.001* vs. PE). This endpoint increase represented a partial structural recovery, with a calculated FRI of 68.3% ± 19.0% (Figure 4B).

### 3.3 Rapid improvement of placental oxygenation and functional perfusion

#### 3.3.1 Placental oxygenation (PAI sO_2_)

**FIGURE 5:**
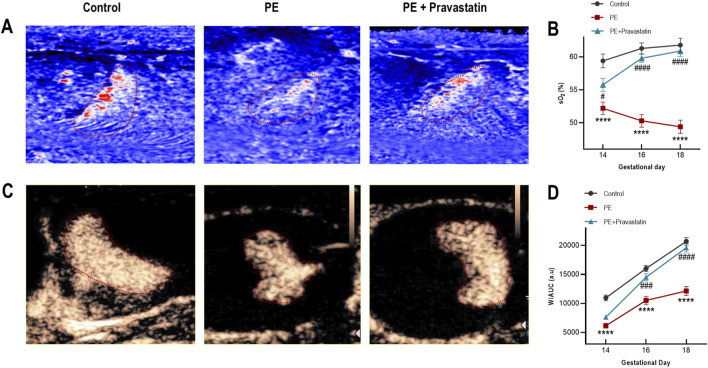
Temporal dynamics of placental oxygenation and functional perfusion. **(A,B)** Placental oxygenation (sO_2_). Representative heatmaps at GD18 **(A)** and longitudinal quantitative profiles **(B)** illustrate the trajectory of oxygen saturation in the pravastatin treatment group relative to PE. **(C,D)** Microvascular blood volume (WiAUC). Representative pseudo-color maps at peak enhancement (30 s) **(C)** and longitudinal trends **(D)** showing changes in WiAUC. Data are presented as mean ± SEM. ****P < 0.0001 vs. control; #*P <* 0.05, ###*P <* 0.001, ####*P <* 0.0001 vs. PE.

In contrast to the delayed recovery of vascular resistance and density indices, placental oxygenation demonstrated an early therapeutic responsiveness. Significant therapeutic effects were detectable as early as GD14, where sO_2_ levels in the Treatment group were statistically higher than in the PE group (*P <* 0.05), 4 days post-intervention. From GD14 to GD18, the Treatment group maintained a continuous upward trajectory, diverging significantly from the progressive hypoxic decline observed in the PE group. By the study endpoint (GD18), sO_2_ levels approached those of the control group. The calculated FRI reached 92.7% ± 6.6%, indicating a substantial rescue of tissue oxygenation status (Figures 5A,B).

#### 3.3.2 Placental perfusion (CEUS WiAUC)

The relative microvascular blood volume (WiAUC) similarly displayed a marked recovery pattern. While the PE group exhibited a suppressed perfusion trajectory, the Treatment group showed a distinct elevation in microvascular perfusion from GD14 to GD18. Statistical divergence from the PE group was established by GD16 (*P <* 0.001), preceding the changes observed in UtA-RI or MV-flow. By GD18, the WiAUC in the Treatment group achieved an FRI of 88.1% ± 6.3%, suggesting that microvascular blood volume was largely restored despite incomplete recovery of structural and hemodynamic indices (UtA-RI and VI) (Figures 5C,D).

### 3.4 Summary of exploratory pharmacodynamic metrics

Table 1 summarizes the Longitudinal Recovery Slope (β) and Functional Recovery Index (FRI) to characterize the temporal kinetics of the therapeutic response *in vivo*. This quantitative integration highlights a differential pharmacodynamic profile across the monitored parameters.

**TABLE 1:** Summary of exploratory pharmacodynamic metrics of pravastatin therapy.

Metric	Slope β (PE)	Slope β (PE + pravastatin)	P-value (slope)	FRI at GD18 (% recovery)	Response pattern
Systemic markers					
SBP (mmHg/day)	+3.91 ± 0.27	+2.15 ± 0.23	<0.001	51.1 ± 6.9	Attenuated progression
Urinary protein (mg/L/day)	+43.84 ± 4.15	+29.55 ± 1.59	<0.01	32.0 ± 4.1	Attenuated progression
Structural and hemodynamic indices					
UtA-RI (day^-1^)	+0.01 ± 0.005	−0.005 ± 0.002	ns	43.9 ± 4.4	No slope alteration
MV-flow VI (%/day)	−1.07 ± 0.15	−0.73 ± 0.14	<0.05	68.3 ± 19.0	*Partial mitigation*
Oxygenation and perfusion					
PAI sO_2_ (%/day)	−0.70 ± 0.11	+1.95 ± 0.12	<0.001	92.7 ± 6.6^††^	*Trajectory inversion*
CEUS WiAUC (a.u./day)	+1454 ± 51	+3259 ± 42	<0.001	88.1 ± 6.3^†^	*Enhanced increase*

Data are presented as Mean ± SEM. Significance for Slope comparison is derived from Analysis of Covariance (ANCOVA) testing for differences in regression coefficients. Differences in FRI were assessed via One-way ANOVA followed by Tukey’s *post hoc* test.

^†^ *P* < 0.05.

^††^ *P* < 0.01 vs. UtA-RI FRI., ns: not significant.

#### 3.4.1 Kinetics (slope)

Pravastatin significantly altered the temporal trajectory of functional markers. Notably, treatment coincided with an inversion of the sO_2_ slope (Slope shift: −0.70 to +1.95%/day, *P <* 0.001) and significantly enhanced the rate of increase in WiAUC (Slope increase: +1454 to +3259 a.u./day, *P <* 0.001). In contrast, the slopes for structural and hemodynamic indices showed distinct behaviors: UtA-RI showed no significant alteration in slope (*P* > 0.05), while MV-flow VI showed only partial attenuation of the decline (*P <* 0.05).

#### 3.4.2 Efficacy (FRI)

Consequently, a heterogeneous recovery pattern emerged at the study endpoint. The FRI for oxygenation and functional perfusion (sO_2:_ 92.7%; WiAUC: 88.1%) was significantly higher than that of macroscopic arterial resistance (UtA-RI: 43.9%; *P <* 0.01). This suggests that, in this model, functional parameters exhibited a greater degree of normalization compared to macroscopic arterial resistance.

### 3.5 Association with fetal outcomes and histological corroboration

#### 3.5.1 Association with fetal outcomes

**FIGURE 6:**
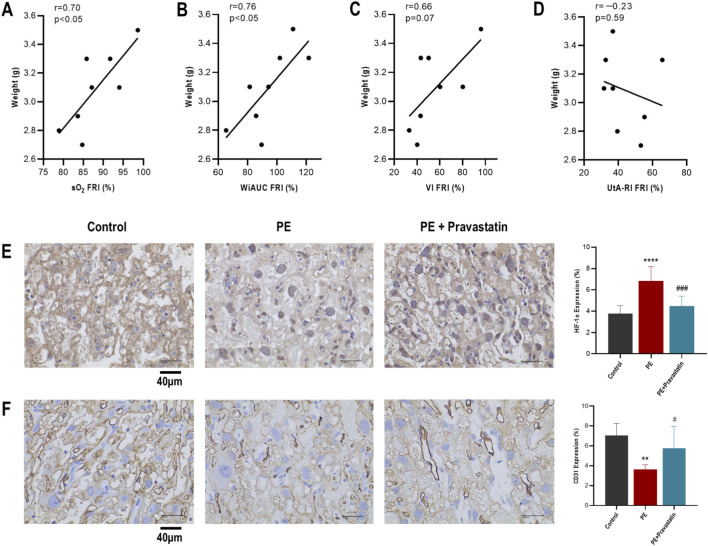
Correlation of functional recovery with fetal growth and histological validation. **(A–D)** Linear regression analysis between fetal weight and specific functional recovery indices (FRIs) in the pravastatin treatment group: **(A)** sO2, **(B)** WiAUC, **(C)** VI, and **(D)** UtA-RI. **(E,F)** Histological validation (scale bars: 40 μm). **(E)** HIF-1α staining confirmed tissue hypoxia status (corroborating sO_2_). **(F)** CD31 staining confirmed microvascular density (corroborating MV-flow). Data are presented as mean ± SEM. **P < 0.01, ****P < 0.0001 vs. control; #*P <* 0.05, ###*P <* 0.001 vs. PE.

To explore the clinical relevance of the observed functional rescue, we analyzed the relationship between the Functional Recovery Indices (FRIs) and fetal weight within the Treatment group. Linear regression analysis revealed that the efficacy of functional rescue was significantly correlated with fetal weight, as evidenced by both metabolic oxygenation (sO_2_: *r* = 0.70, *P <* 0.05) and functional perfusion (WiAUC: *r* = 0.76, *P <* 0.05). In contrast, the FRI of macroscopic arterial resistance (UtA-RI) showed no significant correlation with fetal growth (*r* = −0.23, *P* = 0.59). This dissociation suggests that functional improvements in placental oxygenation and perfusion, rather than the normalization of upstream arterial resistance, are more closely aligned with the therapeutic benefit on fetal growth in this model (Figures 6A–D).

#### 3.5.2 Histological corroboration

To assess the biological consistency of the *in vivo* imaging metrics, a pooled correlation analysis was performed comparing imaging data with *ex vivo* markers. First, regarding oxygenation status, immunohistochemistry revealed a significant inverse correlation between placental sO_2_ measured by PAI and HIF-1α expression (*r* = −0.75, *P <* 0.0001), suggesting that PAI effectively reflected tissue hypoxic status (Figure 6E). Second, regarding microvascular density, CD31 staining corroborated the MV-flow findings. The histological microvessel density showed a significant positive correlation with the VI (*r* = 0.63, *P <* 0.01), supporting the utility of MV-flow as a surrogate for microvascular density (Figure 6F).

## 4 Discussion

In this exploratory study, we utilized a longitudinal multimodal imaging platform to characterize the temporal kinetics of pravastatin treatment in an L-NAME induced rat model of PE. Our data suggest a distinct temporal mismatch between the restoration of placental function and the normalization of vascular architecture. While therapeutic intervention coincided with a rapid improvement in metabolic oxygenation (sO_2_) and microvascular perfusion (WiAUC) as early as GD14, the improvement in macroscopic arterial resistance (UtA-RI) and microvascular density (VI) exhibited a significant lag. In our model, structural recovery remained incomplete despite mid-gestation intervention. This suggests that achieving rapid structural repair in clinical late-gestation scenarios—where the therapeutic window is much shorter—is highly unlikely. Therefore, rescuing placental “functional efficiency” (oxygen extraction and perfusion capacity) represents a more realistic and clinically valuable target.

### 4.1 Mechanisms of rapid functional rescue: the “functional reserve” hypothesis

The rapid improvement in placental oxygenation and perfusion, detectable as early as GD14, suggests a mechanism that may involve acute vascular recruitment rather than immediate angiogenesis (Kingdom and Kaufmann, 1997). Our data revealed a distinct dissociation between functional perfusion and microvascular density: while the WiAUC increased significantly, the VI signal remained comparable to the PE group during this early phase. This pattern suggests that pravastatin treatment coincides with an improvement in functional perfusion efficiency—potentially through the vasodilation of the existing microvascular bed—rather than relying immediately on *de novo* vessel formation.

While molecular verification of the putative eNOS pathway was beyond the scope of this imaging-focused study, the specific pharmacological mechanism of pravastatin provides a compelling explanation for this observed structural-functional mismatch. As a competitive inhibitor of the HMG-CoA reductase enzyme, pravastatin depletes downstream isoprenoid intermediates (e.g., geranylgeranyl pyrophosphate, GGPP). This depletion prevents the membrane translocation and activation of Rho GTPase, thereby inhibiting the Rho/ROCK signaling pathway, which directly leads to the upregulation and stabilization of endothelial nitric oxide synthase (eNOS) (Ahmed and Ramma, 2015; Oesterle et al., 2017). Given the specific vasoconstrictive nature of the L-NAME model (which is fundamentally driven by competitive NOS inhibition), the pravastatin-induced restoration of nitric oxide (NO) bioavailability likely triggers acute vasodilation in the existing placental capillary beds. This molecular cascade neatly explains the swift improvement in functional blood volume (WiAUC) and tissue oxygenation (sO2) observed early in treatment, which occurs independently of, and temporally precedes, the slower, extracellular matrix-dependent process of structural vascular remodeling.

### 4.2 Mechanisms of structural lag: vascular remodeling dynamics

In contrast to the rapid functional response, structural and hemodynamic indices (UtA-RI and VI) appeared less responsive to early-stage intervention. The persistence of elevated UtA-RI until GD18 suggests that the established high-resistance phenotype may be slower to resolve. Our histological observations provide anatomical context for this hemodynamic lag. As shown in Supplementary Figure S4, while pravastatin treatment partially improved trophoblast invasion (CK7), spiral arteries in the PE group retained a persistent smooth muscle layer (high α-SMA) at the study endpoint. This finding is consistent with the understanding that reversing pathological structural remodeling is intrinsically a slower biological process than inducing functional vasodilation (Pijnenborg et al., 2006; Osol and Mandala, 2009). Consequently, it is plausible that while the drug could rapidly mobilize functional reserves, the limited therapeutic window might be insufficient to fully reverse the established structural alterations in spiral arteries (Prefumo et al., 2004).

### 4.3 Translational implications and broader utility

From a translational perspective, our correlation analysis offers preliminary insights suggesting that fetal weight may be more closely associated with functional markers (sO_2_, WiAUC) than with upstream resistance (UtA-RI) in this specific model. This observation parallels clinical findings regarding the limited therapeutic monitoring sensitivity of UtA-RI (Khalil et al., 2010), as macroscopic resistance indices often fail to promptly reflect pharmacological responses. This highlights the potential value of functional biomarkers as complementary endpoints. Beyond the specific evaluation of pravastatin, this multimodal platform could represent a refined preclinical framework for the screening of novel therapeutics (Wareing et al., 2005; George et al., 2013). It may facilitate the evaluation of therapeutic efficacy while phenotypically distinguishing whether a candidate drug acts primarily by dilating existing vessels (increasing WiAUC) or by promoting *de novo* angiogenesis (increasing VI).

Regarding clinical feasibility, CEUS is approaching clinical maturity with an excellent safety profile (Sidhu et al., 2018). However, clinical translation of PAI remains challenged by optical penetration depth (Vincely and Bayer, 2023). Despite these physical barriers, our study highlights the potential value of PAI as a high-throughput tool for preclinical pharmacological research, allowing for the non-invasive monitoring of placental oxygenation in small animal models.

### 4.4 Ethical considerations: application of 3R principles

Consistent with contemporary standards in pharmacological research (Percie du Sert et al., 2020), our study design was guided by the 3R principles. By implementing high-frequency ultrasound for early pregnancy screening at GD7, we excluded non-pregnant animals prior to allocation, thereby optimizing resource use (Reduction) (Hildebrandt et al., 2008). Furthermore, the longitudinal multimodal imaging platform allowed for the serial assessment of placental development within the same subject, eliminating the need for interim euthanasia satellite groups (Refinement) (Tremoleda et al., 2012). This approach exemplifies how advanced imaging can facilitate rigorous pharmacological evaluation while adhering to animal welfare standards.

### 4.5 Limitations

Several limitations warrant consideration. First, the L-NAME model phenocopies endothelial dysfunction but lacks the complex upstream immunological maladaptation of human PE (Bakrania et al., 2022). Second, because our primary objective was to evaluate *in vivo* imaging phenotypes, our biochemical validation was narrowly focused on the core angiogenic axis (sFlt-1/PlGF). We did not collect data on maternal metabolic profiles (e.g., serum HbA1c/glucose or cholesterol/triglycerides), nor did we assess broader endocrine or placental morphogenic biomarkers (such as PAPP-A). Consequently, the precise molecular mechanisms underlying the observed ‘functional reserve’ require future comprehensive biochemical validation. Third, our analysis is inherently restricted to relative longitudinal trends rather than absolute values; this is due to both depth-dependent optical fluence attenuation in PAI (Bauer et al., 2011) and the vasodilatory effects of isoflurane anesthesia, although strict temporal standardization mitigated differential drift. Finally, direct clinical translation of our pravastatin dosing regimen requires caution. Our GD10–18 administration window models mid-to-late gestation, a period when the mature syncytiotrophoblast acts as a lipid barrier; thus, pravastatin’s hydrophilicity restricts passive transplacental diffusion, resulting in minimal fetal exposure as confirmed by clinical umbilical cord blood analyses (Costantine et al., 2016). Although the FDA recently relaxed strict contraindications for statins in high-risk pregnancies (U.S. Food and Drug Administration, 2021), the long-term safety of continuous exposure warrants further investigation.

## 5 Conclusion

In conclusion, this exploratory study indicates that pravastatin therapy may improve placental function prior to complete structural vascular restoration. Our findings suggest that oxygenation and perfusion markers (sO2, WiAUC) may offer earlier insights into therapeutic efficacy compared to macroscopic arterial resistance (UtA-RI) in this model. These observations lend support to the concept of “functional rescue” as a potential therapeutic strategy and highlight the potential utility of multimodal imaging in facilitating the preclinical evaluation of novel placental therapeutics in this experimental setting.

## Data Availability

The raw data supporting the conclusions of this article will be made available by the authors, without undue reservation.

## Supplementary material

SUPPLEMENTARY FIGURE S1 High-frequency ultrasound (HFUS) screening on gestational day 7 (GD7). **(A)** Non-pregnant: representative image showing hypoechoic bands with a central linear endometrium. **(B)** Pregnant: representative image showing multiple gestational sacs (∼2–3 mm) with primitive embryos.

SUPPLEMENTARY FIGURE S2 Flowchart of animal enrollment, randomization, and allocation.

SUPPLEMENTARY FIGURE S3 Longitudinal sonographic monitoring of fetal development. Representative B-mode ultrasound images acquired from gestational day (GD) 7 to GD18, illustrating the normal progression from early gestational sacs (GD7–GD10) to distinct fetal structures (GD12–GD18).

SUPPLEMENTARY FIGURE S4 Histological assessment and quantification of spiral artery remodeling. **(A)** Trophoblast invasion (CK7). Left: Representative images showing reduced invasion in the PE group and partial improvement in the PE + pravastatin group. Right: Quantification of CK7 positive area confirms the histological observations. **(B)** Vascular smooth muscle remodeling (α-SMA). Left: Representative images showing the persistent muscular coat in the PE and PE + pravastatin groups compared to the physiological remodeling (loss of smooth muscle) in the control group. Right: Quantitative analysis of α-SMA expression. Pravastatin treatment significantly attenuated the elevated α-SMA levels observed in the PE group, suggesting an amelioration of pathological vascular remodeling, although the recovery remained incomplete compared to the control group. Data are presented as mean ± SEM. *****P <* 0.0001 vs. control; ##*P <* 0.01, ####*P <* 0.0001 vs. PE. Scale bars: 40 μm.
