# Supplementary figures and images for: In vivo assessment of pravastatin efficacy in an L-NAME rat model of preeclampsia: uncoupling of functional and structural recovery via combined photoacoustic and ultrasound imaging
Source: Front Pharmacol. 2026 May 19;17:1782936. doi: 10.3389/fphar.2026.1782936 (PMC13226026; doi:10.3389/fphar.2026.1782936)

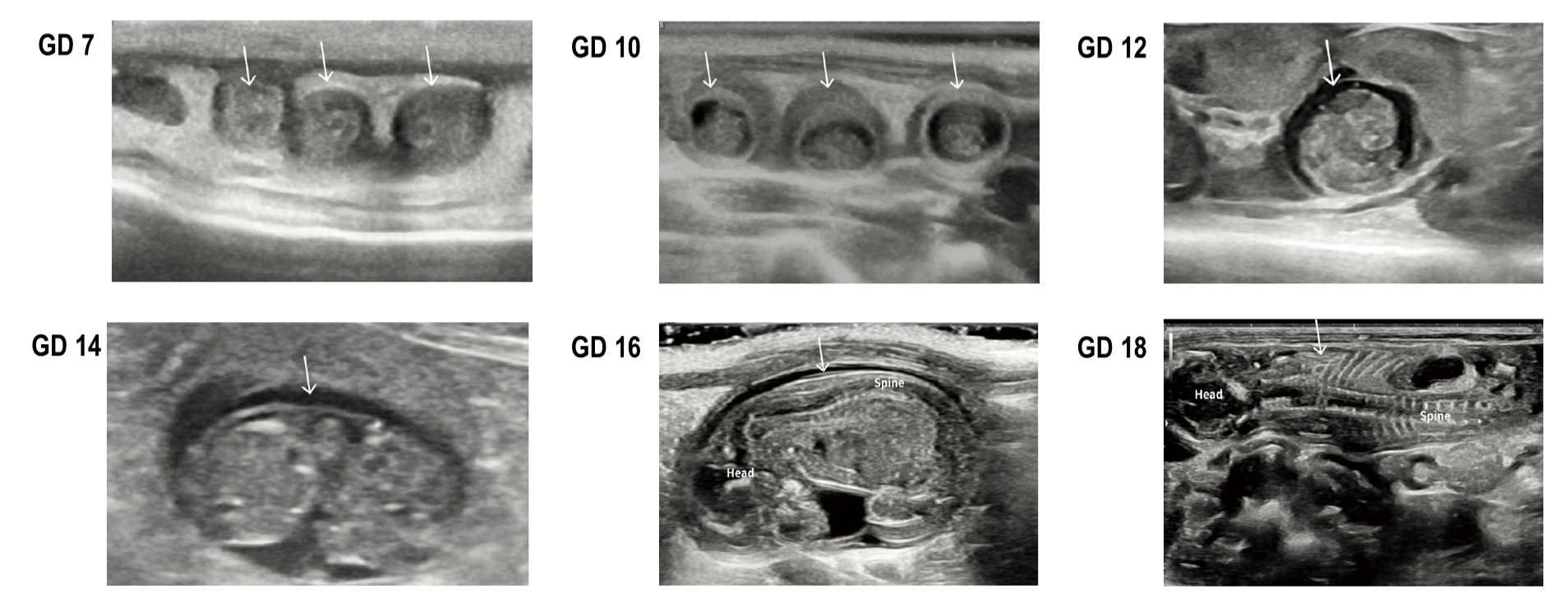

Supplement: Supplementary file 1 [file Image3.tiff]

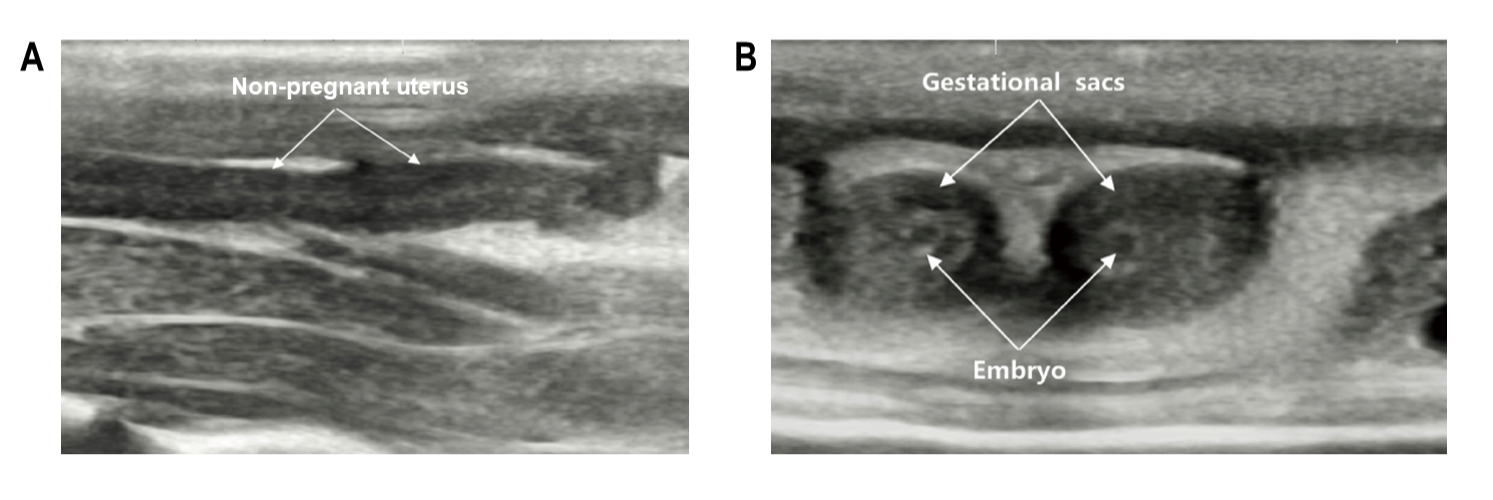

Supplement: Supplementary file 2 [file Image1.tiff]

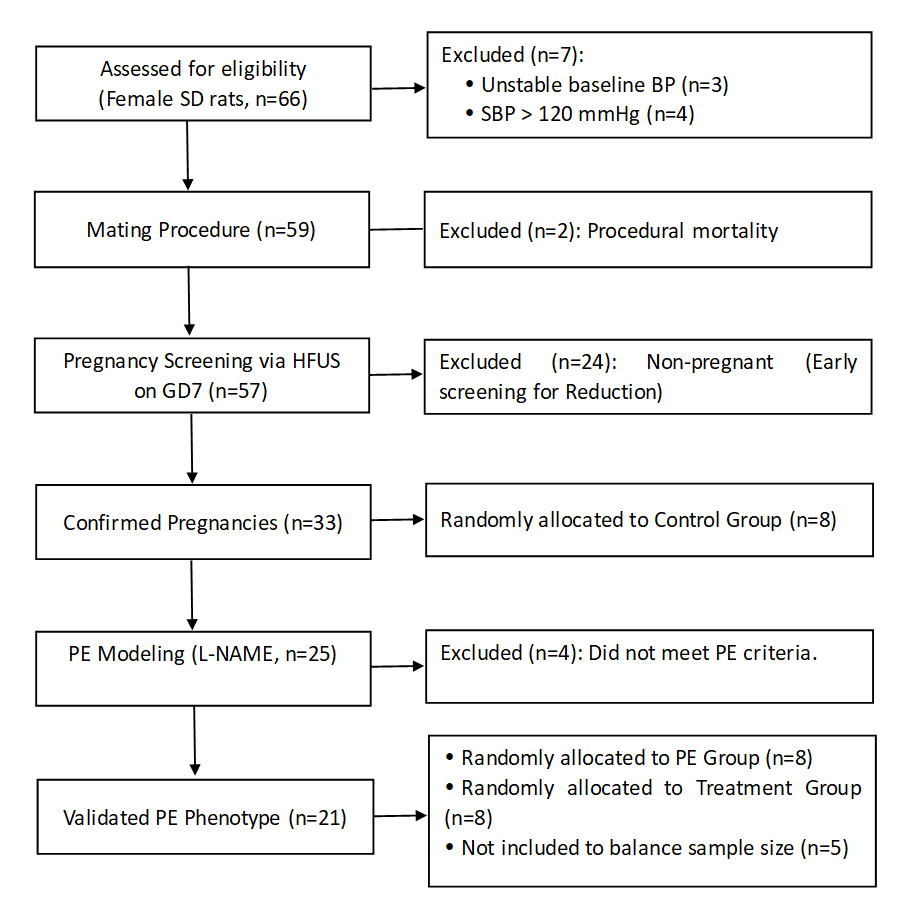

Supplement: Supplementary file 3 [file Image2.tif]

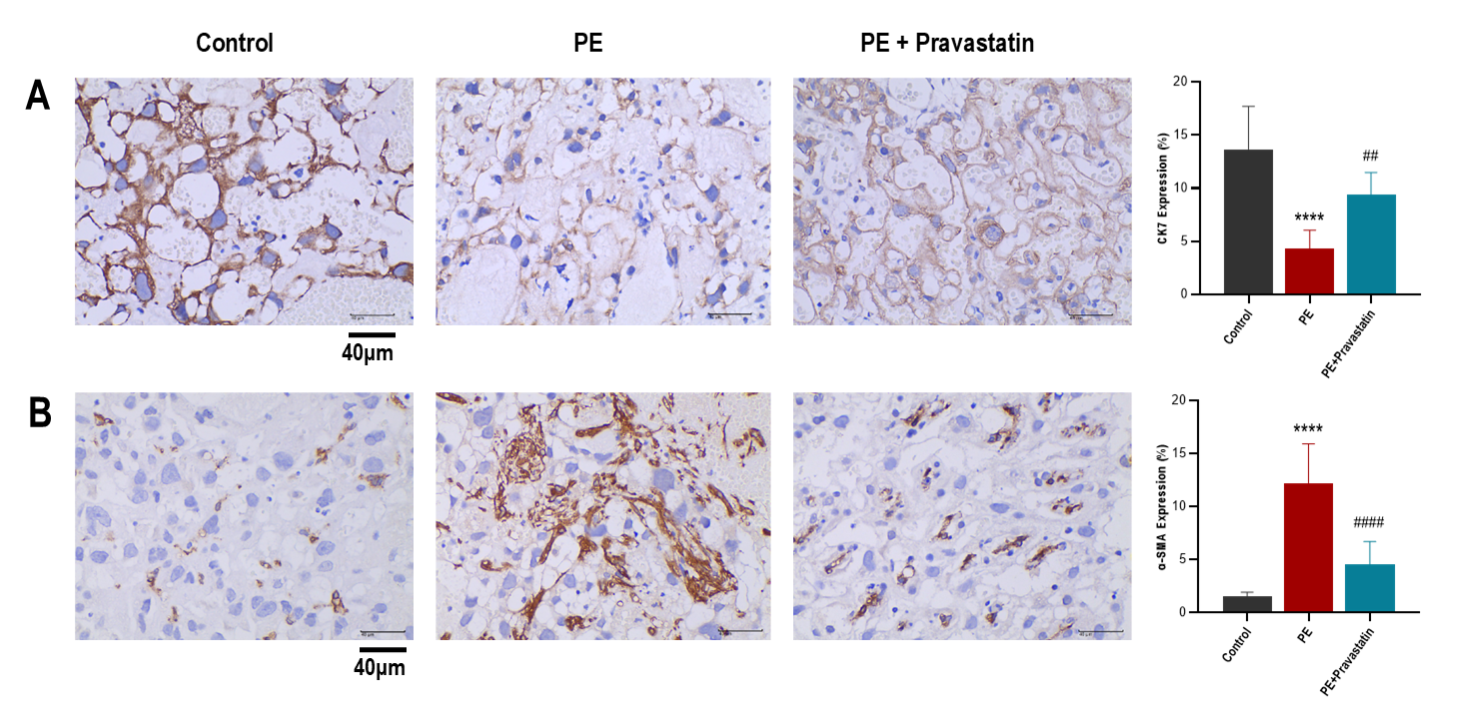

Supplement: Supplementary file 4 [file Image4.tiff]
